# Implementation of marijuana and drug screening with depression and alcohol screening: results from a pilot study integrating behavioral health screening into routine primary care

**DOI:** 10.1186/1940-0640-10-S2-O40

**Published:** 2015-09-24

**Authors:** Gwen Lapham, Megan Addis, Amy Lee, Carol Achtmeyer, Julie Richards, Evette Ludman, Tory Gildred, Ryan Caldeiro, Larry Marx, Paula Lozano, Katharine A Bradley

**Affiliations:** 1 Center of Excellence in Substance Abuse Treatment and Education (CESATE), Veterans Affairs (VA) Puget Sound Health Care System, Seattle, WA, 98108, USA; 2 Department of Health Services, University of Washington, Seattle, WA, 98195, USA; 3 Department of Medicine, University of Washington, Seattle, WA, 98195, USA; 4 Department of Psychiatry and Behavioral Sciences, University of Washington, Seattle, WA, 98195, USA; 5 Group Health Research Institute, Seattle, WA, 98101, USA; 6 Health Services Research & Development (HSR&D) Seattle Center of Innovation for Veteran-Centered and Value-Driven Care, Seattle, WA, 98108, USA

## Background

Routine primary care screening for depression and alcohol use is widely recommended. Benefits of screening for marijuana and drug use are less clear, but might identify patients whose drug use complicates their health. As a result, leaders of a large integrated healthcare system in a U.S. state, where recreational marijuana use was recently legalized, implemented screening for marijuana and drug use as part of a pilot of behavioral health integration in primary care. The objective is to describe the prevalence of screening and further assessment of marijuana and drug use compared to depression and alcohol misuse during early implementation.

## Material and methods

This study included patients who visited the pilot primary care clinic during the initial 6 weeks of implementation. A paper-based 7-item behavioral health screen included the PHQ2 (depression), AUDIT-C (alcohol misuse), and single-item screens for marijuana and drug use. Primary care teams were expected to screen all adults patients annually and further assess positive screens (PHQ2¿2 either item, AUDIT-C&gt;6, daily marijuana use and any drug use) using the PHQ9 (depression) and the assessment of DSM-5 symptoms for alcohol or drug use disorders, respectively. We describe the prevalence of screening, positive screens, and further assessment for each.

## Results

Of 4,332 eligible patients, 76.3% were screened for depression, compared to 74.7% (alcohol), 74.3% (marijuana), and 74.2% (other drug use). Of those screened, 15%, 1.9%, 2.7% and 1.0% were positive for depression, moderate-to-severe alcohol misuse, daily marijuana use and any drug use, respectively. Rates of further assessment of positive screens varied widely: 82.4% for depression, 57.4% alcohol, 37.2% marijuana and 21.2% drug.

## Conclusions

Among these adult Washington State primary care patients seen during pilot implementation, any marijuana use was fairly common (14.1%). Rates of follow-up assessment for those with daily marijuana or any other drug use were lower than for depression and alcohol misuse.

